# Supplementary figures and images for: Analysis of the RNA Editing Sites and Orthologous Gene Function of Transcriptome and Chloroplast Genomes in the Evolution of Five Deutzia Species
Source: Int J Mol Sci. 2023 Aug 19;24(16):12954. doi: 10.3390/ijms241612954 (PMC10454583; doi:10.3390/ijms241612954)

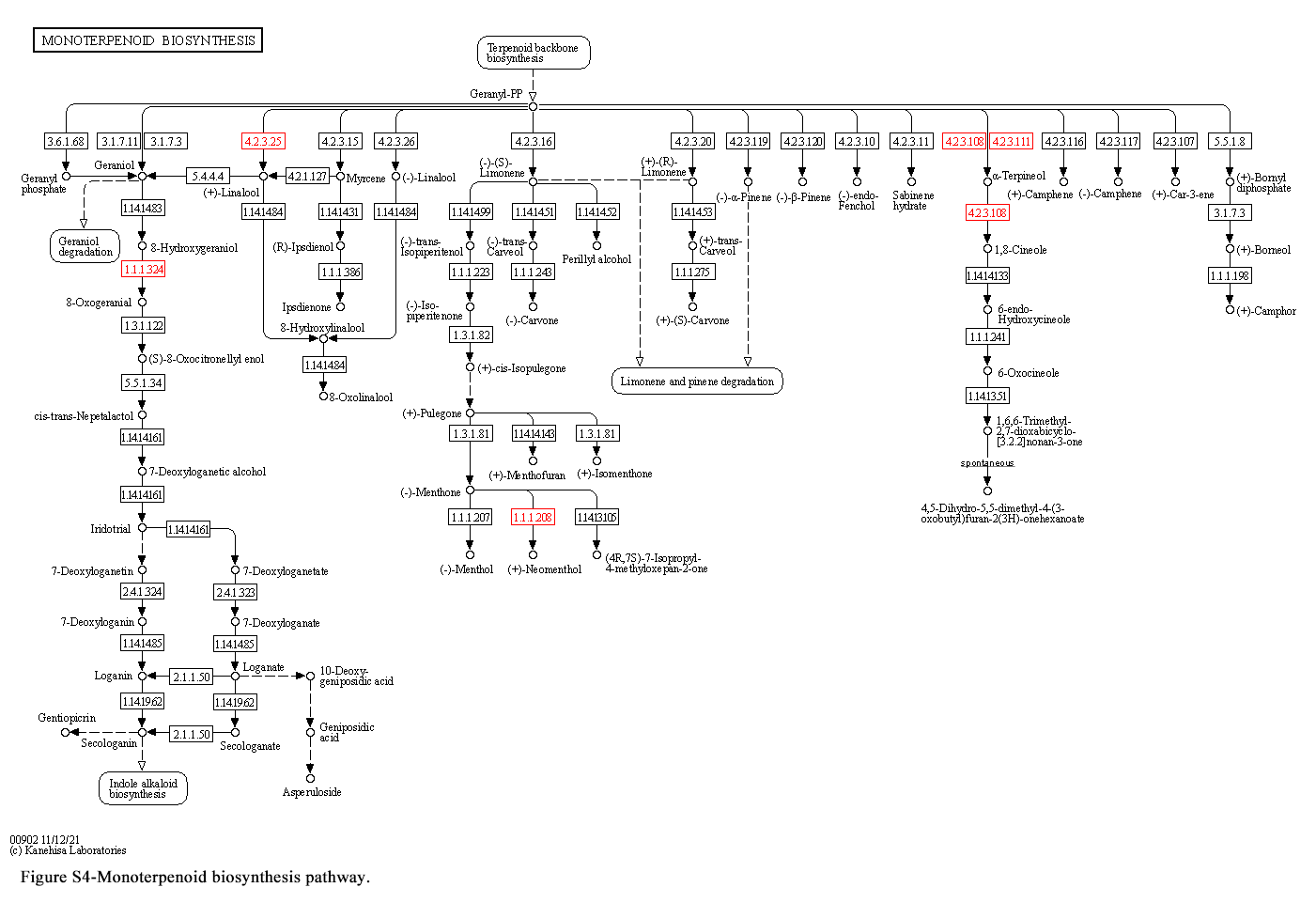

Supplement: Supplementary file 1 [file ijms-24-12954-s001.zip › Figure S4-Monoterpenoid biosynthesis pathway.png]

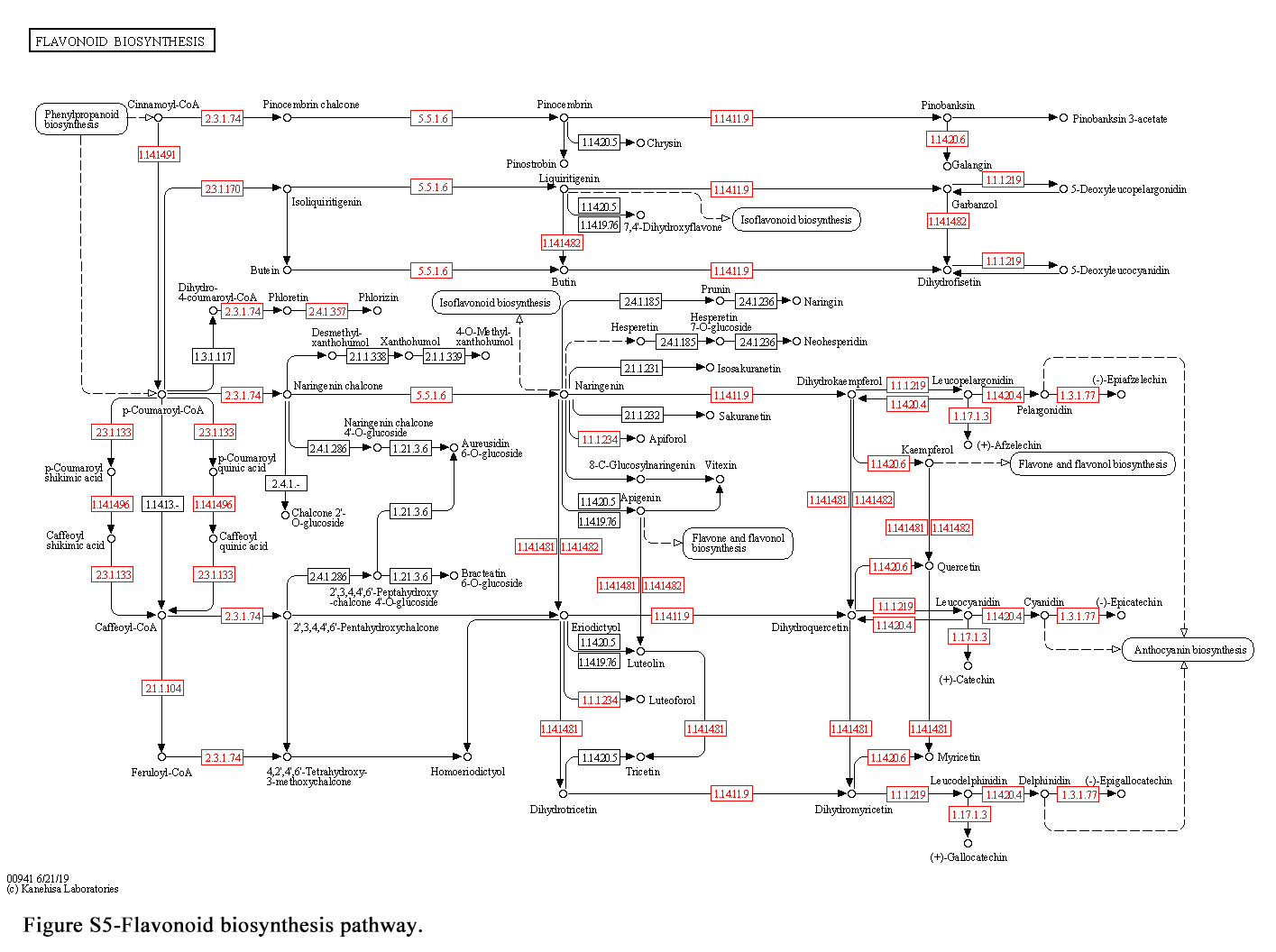

Supplement: Supplementary file 1 [file ijms-24-12954-s001.zip › Figure S5-Flavonoid biosynthesis pathway.png]
